# Comparative genomic analyses of aerobic planctomycetes isolated from the deep sea and the ocean surface

**DOI:** 10.1007/s10482-024-02041-0

**Published:** 2024-11-25

**Authors:** Lise Øvreås, Nicolai Kallscheuer, Rita Calisto, Nicola Bordin, Julia E. Storesund, Christian Jogler, Damien Devos, Olga Lage

**Affiliations:** 1 Department of Biological Sciences, University of Bergen, Bergen, Norway; 2 Department of Microbial Interactions, Friedrich Schiller University, Jena, Germany; 3 Department of Biology, Faculty of Sciences and CIIMAR, University of Porto, Porto, Portugal; 4 Institute of Structural and Molecular Biology, University College London, London, UK; 5 Institute of Marine Research, Bergen, Norway; 6 CABD, Universidad Pablo de Olavidade, Seville, Spain; 7 Centre d’Infection Et d’Immunité de Lille, Institut Pasteur de Lille, University of Lille, Lille, France

**Keywords:** *Planctomycetota*, Deep sea, Iron hydroxide deposits, Surface water, Biofilm, Genome comparison

## Abstract

On the deep and dark seafloor, a cryptic and yet untapped microbial diversity flourishes around hydrothermal vent systems. This remote environment of difficult accessibility exhibits extreme conditions, including high pressure, steep temperature- and redox gradients, limited availability of oxygen and complete darkness. In this study, we analysed the genomes of three aerobic strains belonging to the phylum *Planctomycetota* that were isolated from two deep-sea iron- rich hydroxide deposits with low temperature diffusive vents. The vents are located in the Arctic and Pacific Ocean at a depth of 600 and 1,734 m below sea level, respectively. The isolated strains Pr1d^T^, K2D and TBK1r were analyzed with a focus on genome-encoded features that allow phenotypical adaptations to the low temperature iron-rich deep-sea environment. The comparison with genomes of closely related surface-inhabiting counterparts indicates that the deep-sea isolates do not differ significantly from members of the phylum *Planctomycetota* inhabiting other habitats, such as macroalgae biofilms and the ocean surface waters. Despite inhabiting extreme environments, our “deep and dark”-strains revealed a mostly non-extreme genome biology.

**Supplementary Information:**

The online version contains supplementary material available at 10.1007/s10482-024-02041-0.

## Introduction

The deep sea is the largest ecosystem on Earth and accounts for approximately 75% of the total ocean volume and hosts 62% of the global biosphere (Fang et al. 2010; Kallmeyer et al. 2012). Bacteria inhabiting surface environments and those thriving in the depths of the ocean have diverged over evolutionary time, leading to distinctive genomic adaptations that enable them to exploit the resources and withstand the stresses of their specific ecological niches (DeJong and Karl, 2005; Zhou et al. 2020). Surface-dwelling bacteria typically encounter fluctuating conditions, including variable light, temperature, and nutrient levels, and often rely on photosynthetic energy sources or organic matter derived from terrestrial ecosystems. In contrast, deep-sea bacteria are adapted to a cold, high-pressure, nutrient-scarce, and completely dark environment, where they depend on chemosynthesis or the limited organic matter that sinks from the upper layers of the ocean (Lauro and Barlett, 2008). Surface bacteria frequently harbor genes that confer adaptability to dynamic conditions, such as mechanisms for rapid metabolic shifts, genes for UV resistance, and a broad range of transport systems for varied nutrient sources. By contrast, deep-sea bacterial genomes often reflect streamlined metabolic pathways, adaptations for coping with high hydrostatic pressure, and genes for metabolizing the limited nutrients available in the deep ocean (Oger et al., 2010). Additionally, the relatively stable but extreme conditions of the deep sea may favor genomic traits that promote long-term survival over rapid growth, in contrast to the more opportunistic strategies often observed in surface bacteria. Deep-sea ecosystems are largely unexplored and harbour an untapped diversity of life, including archaea and bacteria (Hoshino et al. 2020; Salazar et al. 2015; Walsh et al. 2016).

A phylum of ubiquitous bacteria is *Planctomycetota* that has attracted the interest of several research groups since the last century (Neef et al. 1998). Members of the phylum are characterized by a complex cell plan and life cycle, unknown secondary metabolite biochemistry and enigmatic genomes with a high percentage of genes with an unknown function (Kallscheuer and Jogler 2021; Rivas-Marín and Devos 2018; Rivas-Marin et al. 2020; Wiegand et al. 2018). Phylogenetically, the phylum is part of the *Planctomycetota*, *Verrucomicrobiota*, *Chlamydiota* (PVC) superphylum (Oren and Garrity 2021; Wagner and Horn 2006). The current taxonomy of the phylum comprises two classes: *Planctomycetia* (Vitorino and Lage 2022), which is the best explored class as assessed by cultivation-dependent and -independent methods, and the less explored class *Phycisphaerae* (Fukunaga et al. 2009). In addition, a third provisional class, *Candidatus* Brocadiia (Lodha et al. 2021), includes bacteria capable of anaerobic ammonium oxidation (“anammox” metabolism) (Strous et al. 1999). Recently, the provisional class *Candidatus* Uabimicrobiia was added after the isolation of two *Candidatus* Uabimicrobium species, exceptional obligatory predatory bacteria capable of phagocytosis-like cell engulfment (Shiratori et al. 2019, Wurzbacher et al. 2024).

All validly described members of the phylum are chemoorganotrophs that occur in a wide range of habitats (Lage et al. 2019). Many strains have been detected in or isolated from aquatic environments, both marine and freshwater, *e.g*. directly from the water column, marine snow, the surface of macroalgae and aquatic animals, and from coastal sediments (Wiegand et al. 2018). However, their occurrence is not limited to aquatic habitats, as they are also found in terrestrial, extreme and polluted environments or associated with various eukaryotes including humans (Cayrou et al. 2013; de Araujo et al. 2021). Despite their ubiquity, they are in most cases not the most abundant phylum. However, high abundances have been reported, for example, in the following habitats: biofilms of macroalgae (Bengtsson and Øvreås, 2010) and seagrass (Kohn et al. 2020a); aridic regions in China (23.7% of the bacterial community (Chen et al. 2017a)); the active layer above permafrost soils on the Tibetan Plateau (Chen et al. 2017b); acidic *Sphagnum* peat bogs and lichen-dominated tundra wetlands (Dedysh and Ivanova 2018; Ivanova and Dedysh 2006); marine snow (Reintjes et al. 2023); the oxygenated hypolimnion of freshwater lakes (Okazaki et al. 2017); and moist acidic tundra soil (Kim et al. 2016). Aerobic and anaerobic members of the two validly published classes have also been isolated from deep sea environments (Storesund et al. 2018; Storesund and Øvreås, 2013; Zheng et al. 2024).

Knowledge on the presence and function of planctomycetes in deep-sea environments is scarce, particularly when compared to shallow and surface waters from which most of the hitherto isolated strains have been obtained. Bacteria belonging to the “anammox group” of the phylum (class *Ca*. Brocadiia) are known to exist in the Black Sea’s suboxic zone (Kirkpatrick et al. 2006; Fuchsman et al. 2012). The diversity of the phylum in two different marine hydrothermal vent deposits, the Mohns Ridge, a part of the Arctic Mid Ocean Ridge (AMOR, 600 m depth) and the Valu Fa Ridge (VFR, 1,734 m depth) in the Southwestern Pacific, was analysed by both cultivation-dependent and -independent approaches (Storesund et al. 2018; Storesund and Øvreås, 2013). Abundances of 10–11% for the phylum *Planctomycetota* were observed in both locations.

Since environmental factors (temperature, availability of light and electron donors, etc.) in the deep sea differ significantly from conditions close to the surface of the water column, these differences might also be reflected in the lifestyle as assessed by alterations in metabolic capabilities. As a starting point to decode the cell biological and metabolic capabilities in the phylum *Planctomycetota*, we performed a comparative analysis of genomic features of three aerobic planctomycetes isolated from the deep sea and close relatives that were retrieved from surface waters.

## Materials and methods

### Isolation and cultivation of three deep-sea planctomycetotal strains

The three deep-sea isolates Pr1d^T^, K2D and TBK1r, were analysed and compared with close relatives from the water surface (Table 1). Strain Pr1d^T^ was collected from iron-hydroxide deposits at 600 m depth from the Mohns Ridge (Storesund and Øvreås, 2013). The temperature at the site was 2 °C in the surrounding seawater and 7 °C 10 cm into the iron hydroxide deposits. The pH was 6.6 in the sampled material. Samples were collected and placed in a container at the bottom of the sea which was closed before it was transported to the surface through the water column. Strains K2D and TBK1r were isolated from iron-hydroxide deposits in the south Pacific Ocean, more specifically from the northern end of the Valu Fa Ridge segment, the Vai Lili vent fields at 1,734 m depth (Storesund et al. 2018). The seawater temperature at the bottom was 2.5 °C. The sample material at this site was fluffier and was therefore collected by a slurp gun by sucking the material into a clean container connected to the Remote Operated Vehicle (ROV). The container was closed before the samples were brought back to the water surface and brought onto the boat. The samples from this site had a lower pH with values > 2.8. It also contained high concentrations of iron and manganese (1000–10 000 µmol/kg and ~ 8 × 10^3^ µmol/kg respectively). Samples were inoculated into various aerobic media for stimulating enrichment of planctomycetotal strains. Strain Pr1d^T^ was grown in M13 medium (Schlesner 1994), prepared in aged 70% (v/v) seawater (sea water kept under dark for at least 8 weeks, (ZoBell 1946)). Strain K2D was cultivated in M30 medium (Schlesner 1994), also prepared in aged 70% (v/v) seawater, whereas strain TBK1r was cultivated by diluting the samples 1:100 before plating directly on gelrite plates containing seawater-peptone-yeast extract (SPYG). A detailed description on the cultivation conditions is given in Storesund and Øvreås (2013) and Storesund et al. (2018). All isolation media contained 200 mg/L ampicillin and the cultures were incubated under aerobic conditions in the dark.

**Table 1:** Information on the isolation and physiological characteristics of the deep-sea strains and close relatives isolated from the water surface. n.d. not determined

Characteristics	*Bythopirellula goksoeyrii*	*Bythopirellula polymerisocia*	*Botrimarina mediterranea*	*Botrimarina mediterranea*	*Stieleria* sp.	*Stieleria* sp.
	Pr1d^T^	Pla144^T^	K2D	Spa11^T^	TBK1r	SV7_m_r
Family	*Lacipirellulaceae*	*Lacipirellulaceae*	*Lacipirellulaceae*	*Lacipirellulaceae*	*Pirellulaceae*	*Pirellulaceae*
Geographic loation	Arctic ocean	Estuary of the Baltic Sea	South Pacific Ocean	Mediterranean Sea	South Pacific Ocean	Sælenvannet lake
Sampling coordinates	71.300000, − 5.783333	54.097000, 12.151000	− 22.214133, − 176.608017	41.663000, 2.910000	− 22.214150, − 176.608017	60.331700, 5.277300
Depth	600 m	surface	1,734 m	surface	1,734 m	7 m
Time of sampling	2006	2014	2009	2014	2009	2014
Environment	Marine	Brackish	Marine	Marine	Marine	Brackish
Habitat	Iron hydroxide deposits	Polyethylene particles	Iron hydroxide deposits	Seawater	Iron hydroxide deposits	Meromictic lake (brackish water)
Relation to oxygen	Aerobic	Aerobic	Aerobic	Aerobic	Aerobic	Aerobic
Isolation	M13 medium gelrite plates	M1H NAG ASW agar plates	M30 medium gelrite plates	M1H NAG ASW agar plates	Seawater, peptone, yeast extract (SPYG) gelrite plates	M30 medium gelrite plates
Temperature range (°C)	10-27	20–30	10-30	10-36	10-30	n.d.
Energy souce	Heterotrophy	Heterotrophy	Heterotrophy	Heterotrophy	Heterotrophy	Heterotrophy

### 16S rRNA gene amplification and sequencing

After three weeks of incubation, biomass of the cultures was collected and prepared for DNA extraction and sequencing. The near full-length sequence of the 16S rRNA gene was amplified using the primer combination A8f and 1542r (Edwards et al. 1989; Lane 1991). Amplification and sequencing were performed as previously described (Storesund and Øvreås, 2013). The PCR products were purified using the Illustra Exostar Kit as described by the manufacturer (USB Corporation) and subsequently sequenced using the Big-Dye.3.1 kit (ABI 3700 PE; Applied Biosystems). Sanger sequencing was performed on separate 16S rRNA gene amplicons, using an ABI3700 sequencing system (Applied Biosystems).

### Genome sequencing and data availability

Genome sequencing of the three isolates was part of a previous study (Wiegand et al. 2020). The sequences of the 16S rRNA genes and genomes are available from GenBank under the following accession numbers: strain K2D: MK554527 (16S rRNA gene), CP036350 (chromosome) and CP036351 (plasmid); strain Pr1d^T^: MK554554 (16S rRNA gene) and CP042913; strain TBK1r: MK554535 (16S rRNA gene) and CP036432. A surface isolate, strain SV_7m_r, was sequenced in addition and included in the comparative genomics analyses. The 16S rRNA gene and genome sequence of this strains are available from GenBank under accession numbers MK554510 and CP036272, respectively (Wiegand et al. 2020). Strain SV_7m_r was isolated from surface water of the brackish lake Sælenvannet (sampling location: 60.332 N 5.277 E). The lake is part of the North Sea fjord system (Nordåsvannet) close to Bergen, Norway.

### Analysis of phylogenetic markers and tree reconstruction

Phylogenetic analyses were performed for the novel isolates and closely related strains belonging to the same respective genus (Table 1). All genomes were retrieved from the NCBI Genbank database. The sequence identities of the 16S rRNA and *rpoB* genes (both used as phylogenetic markers) were assessed via BLASTn (Altschul et al. 1990; Johnson et al. 2008). Average Nucleotide Identity (ANI) values were calculated using CJ Bioscience’s online ANI calculator at the EzBioCloud platform (Yoon et al. 2017). Average Amino Acid Identities (AAI) were obtained with the online All-vs-all ANI/AAI matrix calculator of the enveomics collection using default parameters (Rodriguez-R and Konstantinidis 2016). The percentage of conserved proteins (POCP) was analysed as described (Qin et al. 2014). A multi-locus sequence analysis (MLSA)-based maximum likelihood phylogenetic tree was constructed using autoMLST with 500 bootstrap replicates (Alanjary et al. 2019). The analysis was performed with the autoMLST-simplified-wrapper tool available on GitHub (https://github.com/KatSteinke/automlst-simplified-wrapper). The analysis included the genomes of strains Pr1d^T^, K2D, TBK1r and SV_7m_r along with the reference genomes of strains belonging to the current families *Pirellulaceae and Lacipirellulaceae* (order *Pirellulales*, class *Planctomycetia)*. The genomes of *Gimesia maris* CA11 (GenBank acc. no. GCA_007747015.1), *Rubinisphaera brasiliensis* DSM 5303^ T^ (acc. no. GCA_000165715.3) and *Planctopirus limnophila* DSM 3776^ T^ (acc. no. GCA_000092105.1) (all belonging to the family *Planctomycetaceae*) served as outgroup. The phylogenetic tree was visualized with iTOL v.6 (Letunic and Bork 2021).

### Pangenome construction and analyses of genome-encoded features

The pangenomes were constructed using anvi’o 8 based on the pangenomics workflow described on the anvi’o website (https://anvio.org/learn) (Eren et al. 2021). The “Estimate Metabolism” workflow of anvi’o 8 (Eren et al. 2021) and RAST (Rapid Annotation using Subsystem Technology) (Brettin et al. 2015) were used for the prediction of metabolic pathways and functions. The profiles of putative carbohydrate-active enzymes (CAZymes) were extracted after annotation of the genomes with eggNOG-mapper 2.1.12 (Cantalapiedra et al. 2021). Biosynthetic gene clusters (BCGs) potentially associated with secondary metabolite biosynthesis were analyzed using antiSMASH 7.1.0 with strict detection and all extra features (KnownClusterBlast, ClusterBlast, SubClusterBlast, MIBiG cluster comparison, ActiveSiteFinder, RREFinder, Cluster Pfam analysis, Pfam-based GO term annotation, TIGRFam analysis, TFBS analysis) enabled (Blin et al. 2023, 2021). Metabolic functions related to iron acquisition, iron oxidation or reduction, and siderophore formation were analysed with FeGenie (Garber et al. 2020). The analysis of genes putatively involved in antimicrobial resistance, stress response, and virulence was performed with the NCBI Antimicrobial Resistance Gene Finder Plus (AMRFinderPlus) with the “plus” function enabled (Feldgarden et al. 2021).

## Results and discussion

### Phylogenetic analysis and positions of the strains in the phylogenetic tree

The phylogenetic inference of the three deep-sea isolates Pr1d^T^, K2D and TBK1r was performed based on five phylogenetic markers and the established threshold values for the delineation of species and genera currently used for the phylum *Planctomycetota* (Table S1). The phylogenetic markers included: (1) 16S rRNA gene sequence similarity (genus threshold: 94.5%, species threshold 98.7%) (Yarza et al. 2014), (2) similarity of a ca. 1300 bp partial sequence of the gene *rpoB* encoding the β-subunit of the RNA polymerase (genus threshold range 75.5–78.0%, species threshold: 96.3% Bondoso et al. 2013; Kallscheuer et al. 2020b), (3) ANI (genus threshold: 73.1%, species threshold: 95%) (Barco et al. 2020; Kim et al. 2014), (4) AAI (genus threshold range: 60–80%, species threshold: 95% (Luo et al. 2014) and (5) POCP (genus threshold: 50%, no species threshold) (Qin et al. 2014).

**Fig. 1:**
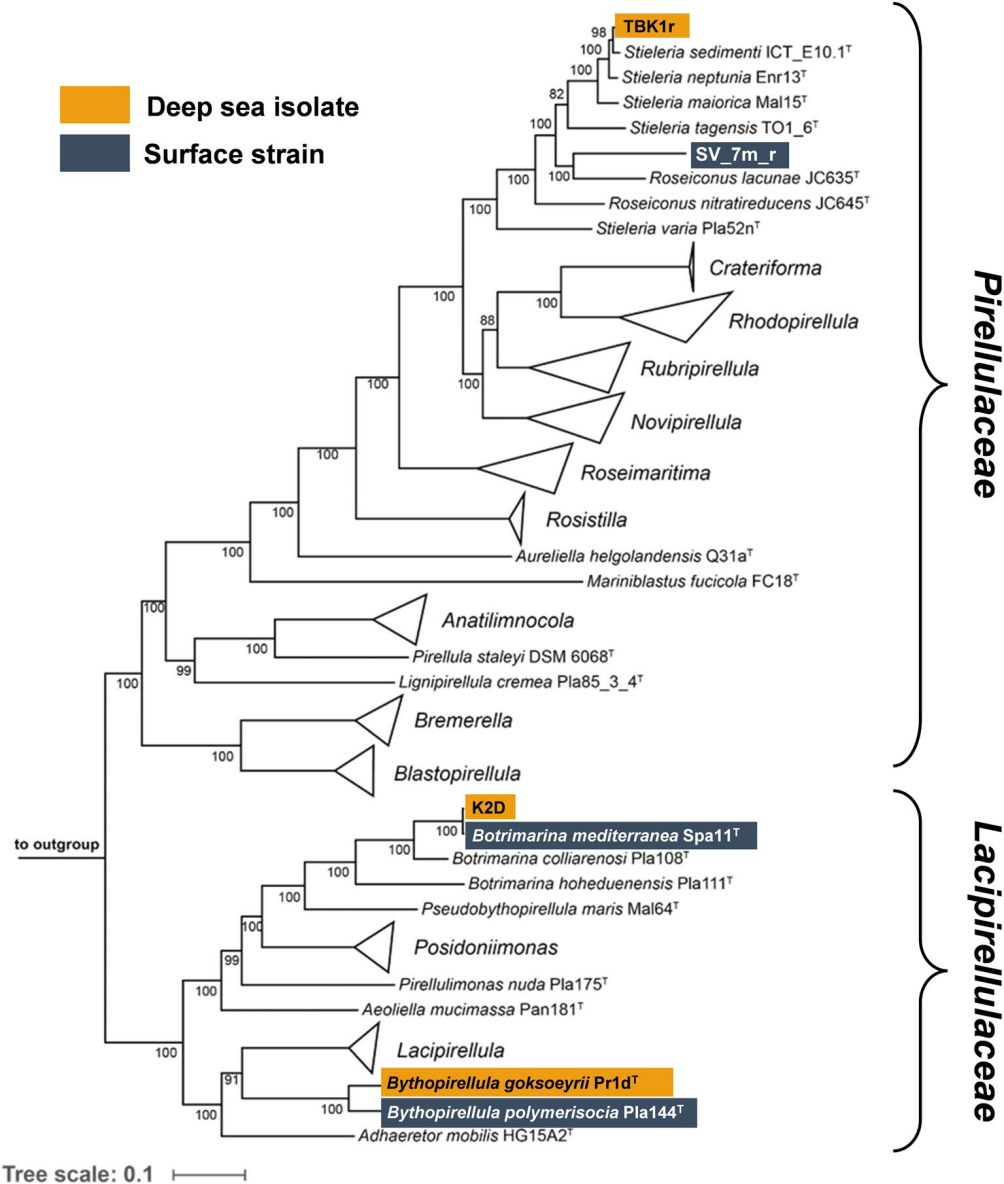
Multi-locus sequence analysis (MLSA)- based phylogenetic tree. The maximum likelihood phylogenetic tree highlights the position of the three deep-sea strains (highlighted in orange). The tree was constructed based on the genomes of all effectively or validly described members of the families *Pirellulaceae* and *Lacipirellulaceae*. The genomes of three members of the family *Planctomycetaceae* were used as outgroup (see Material and methods section for details). Bootstrap values are given at the nodes (in %). The scale bar indicates the number of subtitutions per position. The surface strains that were used for comparison are highlighted in blue

The constructed MLSA-based phylogenetic tree places all three strains in the order *Pirellulales*, more specifically strains Pr1d^T^ and K2D in the family *Lacipirellulaceae* and strain TBK1r in the family *Pirellulaceae* (Fig. 1). The analyzed phylogenetic markers suggest that strain K2D belongs to the already described species *Botrimarina mediterranea* (with type strain Spa11^T^). Strain Pr1d^T^ (the here analysed isolate) was previously validly published as the type strain of the species *Bythopirellula goksoeyrii* (Storesund and Øvreås, 2021) (Table S1). A second member of the genus, *Bythopirellula polymerisocia* Pla144^T^, was isolated from the surface of brackish water in an estuary of the Baltic Sea in Northern Germany (Table 1). The phylogenetic position of strain TBK1r is ambiguous. While the strain is clearly a member of the genus *Stieleria*, the single gene markers (16S rRNA and *rpoB* gene sequence similarity) suggest that the strain belongs to the recently described species *Stieleria sedimenti* (16S rRNA gene sequence similarity: 98.9%, *rpoB* sequence silimarity: 99.5%), whereas the whole genome-based markers ANI and AAI would place it as a novel species (ANI: 91.6%, AAI: 92.1 for comparison of strain TBK1r with *S. sedimenti* ICT_E10.1). In the light of the previously observed low reliability of the species threshold for 16S rRNA gene sequence similarity in the phylum (Kohn et al. 2020b), we give greater weight to the whole genome-based markers and designate the strain *Stieleria* sp. TBK1r.

Despite the isolation from the deep sea, all three strains show close phylogenetic relationship on the level of the same or separate species to already described taxa, for which the respective type strains have all been isolated from surface waters or from abiotic or biotic surfaces in the upper water column. The close relationship and the aerobic lifestyle of all analysed strains facilitates the search for habitat-specific genes that may be required for survival and biomass formation in the respective ecosystems.

### Comparison of genomic features

Basic genomic features of strains Pr1d^T^, K2D and TBK1r were analyzed and compared to close relatives isolated from the water surface (Table 2). The genomes of strains Pr1d^T^ and K2D are similar in size (6.47 and 5.84 Mbp, respectively) and several Mbps smaller than the genome of strain TBK1r (10.77 Mbp). Consequently, the number of genes is also higher in strain TBK1r than in the other two strains. The relative number of genes coding for hypothetical proteins was similarly high; with 41% for Pr1d^T^, 44% for K2D and 46% for TBK1r (based on the automated RefSeq annotation). A high number of proteins with an unknown function has been often observed for members of the phylum *Planctomycetota* (Lage et al. 2019; Overmann et al. 2017) and typically falls between 25 and 45%, depending on the used annotation algorithm and the genome size. The comparison of genomic features of the deep sea isolates and the surface strains only yielded minor differences (reflecting the close phylogenetic relationship), except for the two *Stieleria* strains that showed major differences in size (and consequently numbers of encoded features) and G + C content (Table 2).

**Table 2:** Genomic features of the deep-sea isolates and close relatives isolated from the water surface

Characteristics	*Bythopirellula goksoeyrii*	*Bythopirellula polymerisocia*	*Botrimarina mediterranea*	*Botrimarina mediterranea*	*Stieleria* sp.	*Stieleria* sp.
	Pr1d^T^	Pla144^T^	K2D	Spa11^T^	TBK1r	SV7_m_r
Genome size (bp)	6,473,141	6,143,780	5,839,026	5,871,207	10,769,056	7,107,266
Plasmids	no	inconclusive	1	no	no	no
DNA G + C content (%)	52.8	52.9	64.1	64.1	58.5	55.3
Genes	5107	4902	4609	4549	7611	4991
Protein-coding genes	5007	4794	4516	4484	7337	4848
Protein-coding genes/Mbp	774	780	773	764	681	682
Hypothetical proteins	2036	2020	1973	1925	3393	1876
Hypothetical proteins (%)	40.7	42.1	43.7	42.9	46.2	38.7
Coding density (%)	86.5	86.7	86	85.8	87.4	86.4
CRISPR arrays	1	0	1	0	0	0
tRNA genes	70	79	47	46	106	43
rRNA genes (5S-16S-23S)	1-1-1	1-1-1	1-1-1	1-1-1	2-2-3	2-2-2

### Pangenomics and singleton gene analyses

In the search for genome-encoded features that may reflect the lifestyle in the deep sea, we first compared strains K2D, Pr1d^T^ and TBK1r individually. The comparison was performed against the genomes of all characterized members of the respective genera to which the strains belong, namely *Botrimarina*, *Bythopirellula* and *Stieleria* (cf. Figure 1). The type strains of all described species chosen for comparison were isolated from the surface zone of marine or brackish environments in Europe (North Sea, Baltic Sea, Mediterranean Sea or Atlantic Ocean) or India (Table 1). Based on the pangenomes, singleton genes of the deep sea-originating strains were extracted and analyzed based on their annotation (Tables S2, S3 and S4).

**Fig. 2:**
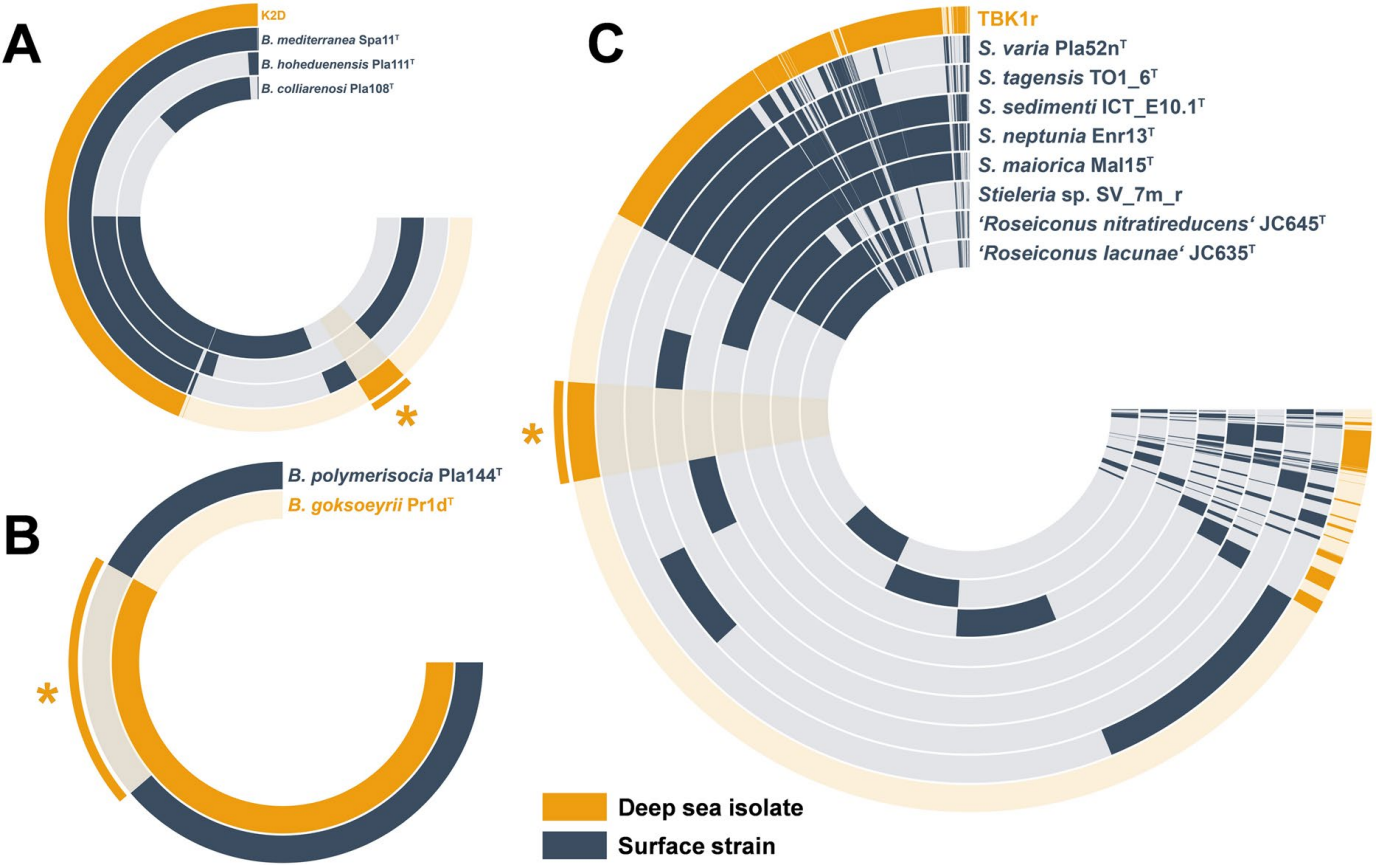
Visualization of the individual pangenomes. **A** Genus *Botrimarina* and strain K2D, **B** Genus *Bythopirellula* and strain Pr1d^T^, C) Genus *Stieleria* and strain TBK1r. Each open circle represents the pangenome of all strains but is colored darker when the gene is present in the respective genome. The analyzed deep-sea strains are shown in orange, all others in blue. The asterisk marks the singleton genes of the respective deep-sea strain

The *Botrimarina* pangenome (based on four genomes) consisted of 7060 clusters, of which 295 were specific for strain K2D (Fig. 2A). After extraction of the annotation information based on NCBI’s Database of Clusters of Orthologous Genes (COG20) and curation of the list by removal of hypothetical proteins and proteins with an unknown function, 84 genes remained (Table S2). In the same manner, pangenomes of the current genera *Bythopirellula* (two genomes, 6547 clusters) and *Stieleria* (including the genus “*Roseiconus*”) (nine genomes, 22,507 clusters) were constructed (Fig. 2B, C). After curation, 747 singleton genes with a putative gene annotation were obtained for strain Pr1d^T^ and 408 for strain TBK1r (Tables S3 and S4). The inspection of the curated lists (with entries of hypothetical proteins removed) did not yield any genes coding for enzymes with primary (metabolic) functions, *e.g.* involved in central metabolism, transcription, translation, amino acid and nucleotide biosynthesis, etc. This can be regarded as a plausibility control for the performed analysis since these genes are expected to fall in the respective core genomes (and were also found therein). However, immediate hits that might indicate a facultatively anaerobic/microaerophilic lifestyle or adaptation to higher concentrations of (heavy) metals expected to be required for survival in the deep sea were not obvious. The lists consisted mainly of strain-specific genes that *e.g.* encode enzymes with regulatory functions (protein kinases, transcriptional regulators, sigma factors), DNA-modifying enzymes (recombinases, transposases, CRISPR-Cas proteins, endonucleases, enzymes of restriction-modification systems), polysaccharide catabolic enzymes (sulfatases, sugar debranching enzymes, glycosyltransferases), transporters and phage proteins and mobile elements. In particular the presence of “selfish” genes of phage origin has been consistently observed in studies of deep-ocean microorganisms (Konstantinidis et al. 2009, Smedile et al., 2013). The maintenance of these genes is assumed to be favored by relaxed purifying selection in deeper waters (Konstantinidis et al. 2009). While identifying functions from the genomic analysis along is difficult, their presence suggests a role in environmental adaptation. The axenic strains are available for more detailed analyses, which can be a decisive advantage over analyses based on metagenome-assembled genomes (MAGs). Many of the putative transporters are annotated as efflux proteins for toxic compounds including heavy metals, however, their exact function cannot be derived from the genome information only.

**Fig. 3:**
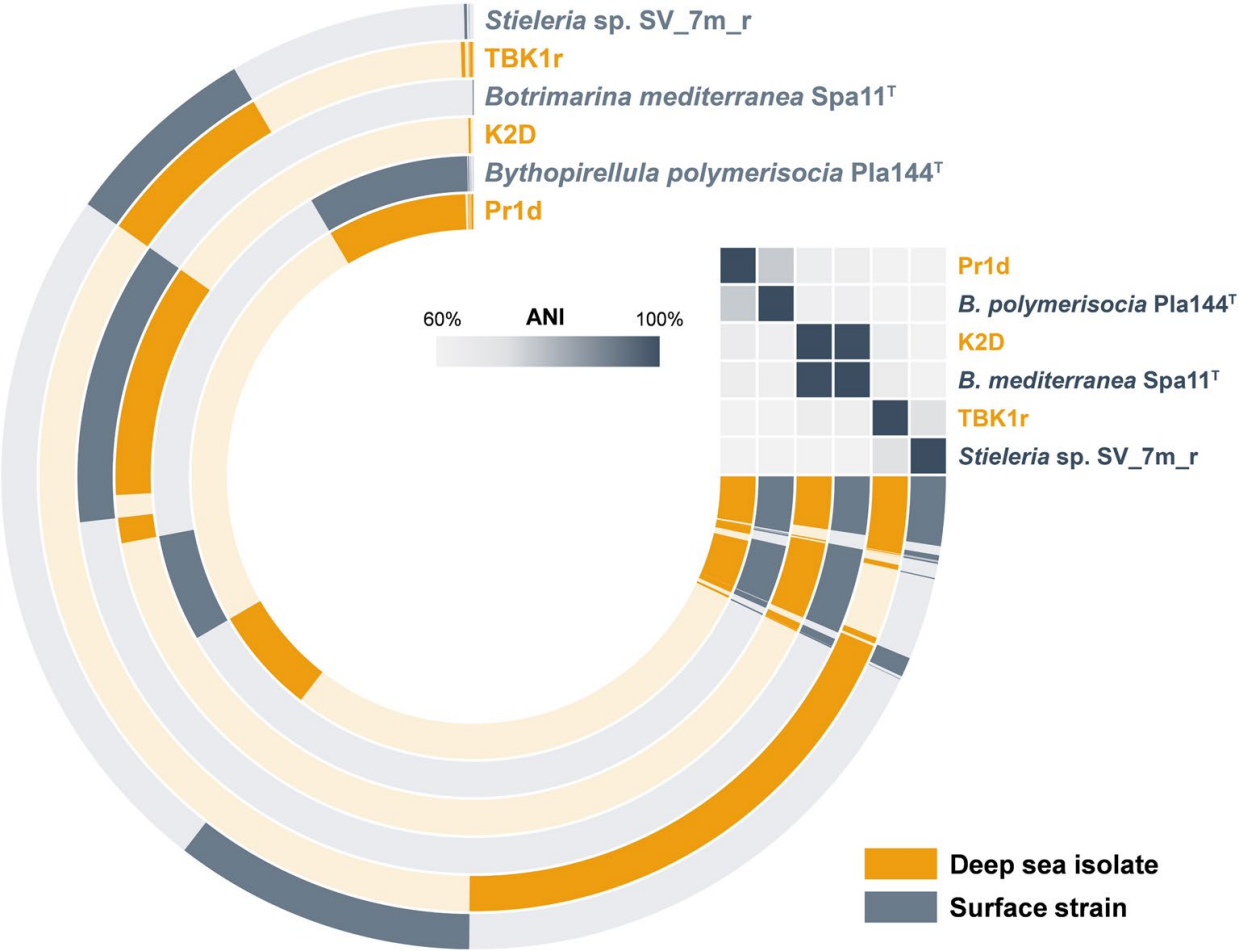
Visualization of the combined pangenome. The open circle depicts the pangenome of the three deep-sea strains (in orange) and a respective close relative from the same genus obtained from surface water (in blue). Each open circle represents the pangenome of all strains but is colored darker when the gene is present in the respective genome. The heatmap in the upper right corner shows the phylogenetic relationship based on average nucleotide identity (ANI) values

In order to check for the presence of conserved deep-sea specific genes, a combined pangenome of the three deep-sea isolates and their respective next relatives was constructed in a second approach (Fig. 3). For strain TBK1r, *Stieleria* sp. SV_7m_r, a free-living isolate from surface water of a meromictic lake was used, since many of the other close relatives were either isolated from non-natural abiotic surfaces or from sediments or lack complete genome sequencing data (assembly level “contigs” or “scaffolds”). Strain Pr1d^T^ only has one closest relative belonging to the same genus, *Bythopirellula polymerisocia*, whereas strain K2D belongs to the already validly published species *Botrimarina mediterranea* (Fig. 1). The obtained combined pangenome did not reveal a conserved set of genes that is absent in the surface strains (Fig. 3). Since the analysed strains belong to two different families, the phylogenetic distance might be already too large for yielding reliable results. The shared genes in the pangenome reflect the closer phylogenetic relationship (9–12 o’clock in the pangenome visualization in Fig. 3) within the genus boundaries. Unfortunately, the analysis did not reveal additional candidates specifically present in the deep sea isolates.

### Analysis of plasmid-encoded genes in strain K2D

The deep-sea strain K2D harbours a 70 kb plasmid with 65 predicted open reading frames that is absent in the surface strain Spa11 (belonging to the same species). 61 of these plasmid-encoded genes turned out the be singletons that were also detected in the pangenome analysis. The plasmid-encoded nature of these singleton genes can provide additional support for specialized functionalities associated with the presence of this extrachromosomal element in strain K2D. Indeed, the automated annotation of several of the plasmid-encoded proteins suggests a role in heavy metal resistance, e.g. including putative subunits of a cobalt-zinc-cadmium efflux protein (CzcABC) and cobalt-zinc-cadmium: H^+^ /K^+^ antiporter (CzcD) along with putative mercuric reductase (MerA), cadmium-transporting ATPase (CadA) and ferrous iron efflux protein F. The genes are organized as a “heavy metal resistance genomic island” between kilobase positions 38 and 55 relative to the replication initiator protein-encoding gene (rotated to position 1).

### Genome-based estimation of metabolic pathways

The “Estimate Metabolism” workflow (of anvi’o 8) was used to assign proteins encoded by the three deep-sea isolates to primary metabolic pathways based on KEGG pathway modules. For comparison, the genomes that were also used for the individual pangenome analyses were included. The lists with complete modules (> 75% of the required enzymes per pathway present) for all analyzed strains were concatenated and inspected for pathways specific to the deep-sea isolates (Table S5). Except for differences in the completeness of some biosynthetic pathways for amino acids and vitamins, no pathways exclusively present in the deep-sea strains were obtained. All isolates including the three aerobic deep-sea strains harbour the genes coding for the subunits of the cytochrome c oxidase catalyzing the terminal oxygen-dependent step. The same is true for the light-dependent DNA photolyase. None of the strains harbours rhodopsin-encoding genes.

In a separate analysis, an annotation using the RAST server was performed for all six genomes. The above-mentioned strain pairs (A: deep-sea isolate, B: surface isolate) were compared using the “Function-based comparison” tool of the SEED-Viewer. The predicted functions present in strain A and absent in B, and the other way around (absent in A and present in B) were collected (Table S6). For the *Botrimarina* strain pair, four protein functions were specific to strain K2D and seven to strain Pla144 (Table S6A, B). These include reactions involved in amino acid and vitamin biosynthesis (cysteine, histidine, folate) and DNA-binding and/or -modifying enzymes (CRISPR-Cas proteins, restriction modification system, transcriptional regulators). A comparison of the *Bythopirellula* spp. pair yielded 32 specific hits each for both analyzed genomes (Table S6C, D). The respective functions comprise amino acid and cofactor biosynthesis, nitrogen metabolism and various electron transfer and transport processes. The largest differences were obtained for the two compared *Stieleria* spp. 125 proteins were predicted to be specific for the deep-sea strain TBK1r and 42 for the surface isolate SV_7m_r (Table S6D, E). The data suggest the absence of the NADH:ubiquinone oxidoreductase NDH-1 (complex I of the respiratory chain) in the surface strain SV_7m_r. This finding was confirmed with the genome annotation obtained from eggnog-mapper that yielded the respective genes (*nuoA-nuoN*) in strain TBK1r, but only *nuoL* in strain SV_7m_r. The complete set of *nuo* genes was also detected in the draft genome of *Stieleria sedimenti* ICT_E10.1. The transfer of electrons from NADH is probably taken over by the NADH:ubiquinone oxidoreductase NQR that is coupled to the transport of Na^+^ ions from the cytoplasm to the periplasm. The respective genes (*nqrA-F*) could be identified in all six analysed genomes. Genes encoding an Na^+^/H^+^ antiporter consisting of seven different subunits were also absent from the genome of strain SV_7m_r, but encoded in strain TBK1r. Several proteins involved in partial steps of cobalamin (vitamin B12) biosynthesis were among the functions predicted to be present in strain SV_7m_r but absent in strain TBK1r.

In a more targeted search, genes involved in common fermentation pathways and nitrate respiration were analyzed in the six genomes (Table 3). Each of the six genomes harbours a lactate dehydrogenase-encoding gene (*Idh* or *IdhA*) that should allow the formation of lactate from pyruvate. Genes encoding enzymes involved in acetate formation from acetyl-CoA (phosphotransacetylase and acetate kinase) were found in four out of six strains. A reductive tricarboxylic acid cycle seems to be absent from all strains since genes encoding the three key enzymes fumarate reductase, 2-oxoglutarate synthase and ATP citrate lyase were not detected. Only the two *Botrimarina* strains harbour a putative phosphoenolpyruvate carboxylase gene. The surface isolate *B. polymerisocia* Pla144 is the only of the compared strains that harbours a gene set for a respiratory nitrate reductase. Putative nitrite reductase-encoding genes were predicted in *B. goksoeyrii* and the two *Stieleria* spp. As suggested by the automated genomic comparison with anvi’o and RAST, the three strain pairs show only minor differences regarding genes involved in fermentation and nitrate respiration pathways that are apparently independent of the strains’ origin (surface or seafloor).

**Table 3:** Presence or absence of genes coding for enzymes involved in fermentation pathways and nitrate respiration. The analysis is based on the annotation of the analyzed strains using eggnog-mapper. NCBI accession numbers are provided in case that the enzyme is present

Enzyme	E.C. number	*Bythopirellula goksoeyrii*	*Bythopirellula polymerisocia*	*Botrimarina mediterranea*	*Botrimarina mediterranea*	*Stieleria* sp.	*Stieleria* sp.
		Pr1d^T^	Pla144^T^	K2D	Spa11^T^	TBK1r	SV_7m_r
Fermenation pathways							
L–lactate dehydrogenase	1.1.1.27	no	no	QDV79991.1	QDV75322.1	QDV84145.1	QDT61696.1
D–lactate dehydrogenase	1.1.1.28	QEG36332.1	TWU24790.1	no	no	no	no
Phosphotransacetylase	2.3.1.8	no	TWU24779.1	QDV80294.1	QDV75658.1	QDV81448.1	no
Acetate kinase	2.7.2.1	QEG35494.1	TWU24780.1	QDV80295.1	QDV75659.1	QDV81447.1	QDT60495.1
Reductive TCA cycle							
Phosphoenolpyruvate carboxylase	4.1.1.31	no	no	QDV77716.1	QDV73143.1	no	no
Fumarate reductase	1.3.1.6	no	no	no	no	no	no
2-Oxoglutarate synthase	1.2.7.3	no	no	no	no	no	no
ATP citrate lyase	2.3.3.8	no	no	no	no	no	no
Nitrogen metabolism							
Respiratory Nitrate reductase	1.7.5.1	no	TWU21779.1–TWU21782.1	no	no	no	no
Nitrite reductase	1.7.1.15	QEG37230.1	no	no	no	QDV86603.1	QDT58782.1

### Carbohydrate-active enzymes

Carbohydrate-active enzymes (CAZymes) are classes of proteins involved in the synthesis, modification or degradation of complex polysaccharides (Sun et al. 2023; Wecker et al. 2010). Members of the phylum *Planctomycetota* thrive on the surface of photosynthetically-active primary producers and have been recognized as important part of bacterial communities during the late decay stage of macroscopic phototrophs (Kallscheuer et al. 2021; Zhang et al. 2024). Hence, we checked for differences in the numbers of CAZyme genes in the surface and deep-sea isolates. The compared strains harbour between 8–13 CAZyme-encoding genes per Mbp and showed similar CAZyme profiles in the direct comparison between the closely related isolates (Table 4). Noticeable is the lack of polysaccharide lyase genes in the deep-sea strains, while one putative gene was found in each of the three strains isolated from the surface. However, more deep-sea strains are required to check if this observation is consistent, as with the small sample size the correlation could also be purely coincidental. For the *Stieleria* strains, TBK1r stood out as its genome encodes approximately twice as many glycoside hydrolases, glycosyltransferases and enzymes with carbohydrate-binding modules as the genome of the compared close relative strain SV_7m_r.

**Table 4:** Numbers of genes encoding carbohydrate-active enzymes (CAZymes) and predicted secondary metabolite-associated biosynthetic gene clusters

Characteristics	*Bythopirellula goksoeyrii*	*Bythopirellula polymerisocia*	*Botrimarina mediterranea*	*Botrimarina mediterranea*	*Stieleria* sp.	*Stieleria* sp.
	Pr1d^T^	Pla144	K2D	Spa11	TBK1r	SV_7m_r
Genome size (Mb)	6.47	6.14	5.84	5.87	10.77	7.11
CAZymes						
Glycoside hydrolases	46	46	44	49	35	20
Glycosyltransferases	17	15	22	24	40	21
Polysaccharide lyases	0	1	0	1	0	1
Carbohydrate esterases	2	1	1	1	1	2
Carbohydrate-binding modules	2	2	3	3	8	4
Auxiliary activities	0	0	0	0	0	0
Total	67	65	70	78	84	48
CAZyme genes / Mbp	10	11	12	13	8	7
Biosynthetic gene clusters						
type I PKS	1	1	1	1	1	0
mixed type I PKS-NRPS	0	0	0	0	1	0
type III PKS	0	0	1	1	1	1
*N*–acyl amino acid	0	0	1	1	2	1
NRPS-like	1	1	1	1	2	1
betalactone	1	1	1	1	0	0
other	1	1	1	1	0	0
Non-alpha poly-amino acids	1	0	0	0	0	0
*N*–acetyl–Gln–Gln amide	1	1	0	0	0	0
arylpolyene	0	1	0	0	0	0
lanthipeptide	0	0	0	0	1	0
ectoine	1	0	0	0	0	0
terpene	1	1	0	0	2	2
Total	8	7	6	6	10	5
BGCs / Mbp	1.2	1.1	1.0	1.0	0.9	0.7

### Secondary metabolism-associated biosynthetic gene clusters

Genome mining of planctomycetal genomes using antiSMASH yielded 1–2 biosynthetic gene clusters (BGCs) potentially associated with the production of secondary metabolites (Kallscheuer and Jogler 2021; Wiegand et al. 2020). The relevance of such clusters in the phylum has so far been linked to the biosynthesis of carotenoids, *N*-acylated amino acids and phenolic compounds (Kallscheuer et al. 2020a; Milke et al. 2024; Panter et al. 2019; Santana-Molina et al. 2022). Most of the predicted clusters have not yet been linked to actual compounds. The here investigated strains harbour 5–10 BGCs predicted by antiSMASH. While the two *B. mediterranea* strains were indistinguishable in their BGC profile, only slight differences were obtained for the other two genera.

### Analysis of proteins putatively involved in iron homeostasis

To identify genes coding for proteins involved in iron homeostasis (transport, oxidation/reduction and storage), the genomes were analysed based on the entries of the FeGenie database (Table 5). The results revealed that no genes for iron reduction or iron oxidation were found in any of the isolates. However, genes related to iron transport, siderophore synthesis, transport, and gene regulation are present. Also, genes encoding putative iron storage proteins were obtained in all isolates. However, these genes were also found to be present in the analysed strains isolated from the water surface in similar numbers (Table 5), indicating that these genes are probably not correlated with the environmental conditions of the isolates from the deep-sea environment.

**Table 5:** Results of the FeGenie analysis of the three deep-sea strains and close relatives isolated from the water surface

Protein function	*Bythopirellula goksoeyrii*	*Bythopirellula polymerisocia*	*Botrimarina mediterranea*	*Botrimarina mediterranea*	*Stieleria* sp.	*Stieleria* sp.
	Pr1d^T^	Pla144	K2D	Spa11	TBK1r	SV_7m_r
Iron transport	5	5	4	4	4	6
Heme transport	0	0	0	0	0	0
Heme oxygenase	0	0	0	0	0	0
Siderophore synthesis	0	0	0	0	0	0
Siderophore transport	0	3	0	0	5	4
Siderophore transport potential	8	8	11	11	14	11
Iron-dependent gene regulation	22	21	28	28	28	19
Iron oxidation	0	0	0	0	0	0
Iron reduction	0	0	0	0	0	0
Iron storage	4	2	1	1	3	3
Magnetosome formation	0	0	0	0	0	0

### Prediction of genes involved in stress-response

NCBI’s AMRFinderPlus was used to analyse the genomes for genes involved in antimicrobial resistance, virulence and stress responses including heavy metal tolerance (Tables S7, S8 and S9). The tool predicted several genes that might be involved in the resistance against antibiotics and heavy metals (arsenic, copper, nickel, cadmium and silver), however, most of these were equally detected in both genomes of the respective strain pairs. The deep-sea strain TBK1r was enriched in putative stress response genes. These included genes encoding the Ag^+^-translocating *P*-type ATPase SilP (silver stress) and CopR-like transcriptional regulators along with CopA (copper-resistance protein, laccase-like oxidase).

## Conclusions

In this study, we performed genome-based analyses of the three aerobic strains Pr1d^T^, K2D and TBK1r that were obtained from the deep sea. Biomass production at the seafloor at about 600 or 2,000 m below sea level requires a source of organic matter (OM) that can be used as carbon and energy source. OM is typically synthesized in the surface layers of the oceans by photosynthetic organisms (primary producers) and part of this material sinks and can reach the seafloor where it can feed the biota of the deep ocean (Kirchman 2018). Deep-sea bacteria may also derive carbon from chemoautotrophic microorganisms that oxidize inorganic chemical substances like iron as sources of energy and fix carbon dioxide in the hydrothermal vent system (Dick 2019).

Our analysis points towards a heterotrophic lifestyle like that of strains thriving in surface ecosystems. Isolates found in the deep sea may well be passively transported on sinking particles from the surface (Mestre et al. 2018). We cannot rule out such as scenario for the three here presented isolates, which is in line with the recent finding that members of the phylum *Planctomycetota* are more widespread in surface ecosystems (Ruff et al. 2024). Still, the three isolates need to ensure propagation or at least survival and persistence in the deep sea environment. The analysis of individual pangenomes revealed singleton genes of potential phage origin or with regulatory functions that are commonly enriched in deep sea bacteria (Konstantinidis et al. 2009). In particular the maintenance of a plasmid harbouring a heavy metal resistance-related genomic island in strain K2D supports additional functionalities towards heavy metal resistance in this strain. The availability of all strains in axenic cultures is crucial for phenotypic analyses in future studies, a decisive advantage over genome analyses based on assembled metagenomes.

In the laboratory, all three strains were isolated under aerobic conditions. Hence, the isolation strategy is biased towards strains that can grow in the presence of atmospheric O_2_ levels and the isolates are not necessarily representative for the typical lifestyle or microbial community compositions observed in deep-sea iron deposits. Still, the isolation of closely related strains from the deep sea and the surface of the water column is an indication of a broader metabolic versatility of members of the phylum, especially when regarded in the context of the large genomes and the high number of proteins with an unknown function. Analyses based on strains with a for the most part uncharacterized central metabolism and 40% of the annotated proteins being of unknown function are challenging. Since the analyses were performed with state-of-the art bioinformatic tools and most recent database versions, additional planctomycetal functionalities are beyond what is accessible with current prediction algorithms. Despite these limitations, the analyses yielded a list of candidate genes involved in stress response and related regulatory functions that need to be analysed in the context of planctomycetal lifestyles and growth profiles in greater detail. The relatively slow growth observed for members of the phylum (with typical generation times between 10 and over 100 h under laboratory-scale cultivation conditions) may be a generalist strategy allowing the survival under different environmental conditions.

Axenic cultures of the presented isolates are a contribution towards understanding life in an environment that challenges our knowledge due to remote and almost inaccessible locations and unculturability of the microbiota (Dick 2019).

## Supplementary Information

Below is the link to the electronic supplementary material.

Supplementary file1 (XLSX 16 KB)

Supplementary file2 (XLSX 15 KB)

Supplementary file3 (XLSX 47 KB)

Supplementary file4 (XLSX 30 KB)

Supplementary file5 (XLSX 55 KB)

Supplementary file6 (XLSX 26 KB)

Supplementary file7 (XLSX 30 KB)

## Data Availability

GenBank accession numbers of the analysed genomes are provided in the Materials and Methods section.
